# Rainfall and other meteorological factors as drivers of urban transmission of leptospirosis

**DOI:** 10.1371/journal.pntd.0007507

**Published:** 2022-04-11

**Authors:** Marcelo Cunha, Federico Costa, Guilherme S. Ribeiro, Marilia S. Carvalho, Renato B. Reis, Nivison Nery Jr, Lauren Pischel, Edilane L. Gouveia, Andreia C. Santos, Adriano Queiroz, Elsio A. Wunder Jr., Mitermayer G. Reis, Peter J Diggle, Albert I. Ko

**Affiliations:** 1 Escola Nacional de Saúde Pública, Fundação Oswaldo Cruz, Ministério da Saúde, Rio de Janeiro, Brazil; 2 Instituto de Pesquisas Gonçalo Moniz, Fundação Oswaldo Cruz, Ministério da Saúde, Salvador, Brazil; 3 Instituto de Saúde Coletiva, Universidade Federal da Bahia, Salvador, Brazil; 4 Faculty of Health and Medicine, University of Lancaster, Lancaster, United Kingdom; 5 Department of Epidemiology of Microbial Diseases, School of Public Health, Yale University, New Heaven, Connecticut, United States of America; 6 Faculdade de Medicina, Universidade Federal da Bahia, Salvador, Brazil; Johns Hopkins University Bloomberg School of Public Health, UNITED STATES

## Abstract

**Background:**

Leptospirosis is an important public health problem affecting vulnerable urban slum populations in developing country settings. However, the complex interaction of meteorological factors driving the temporal trends of leptospirosis remain incompletely understood.

**Methods and findings:**

From March 1996—March 2010, we investigated the association between the weekly incidence of leptospirosis and meteorological anomalies in the city of Salvador, Brazil by using a dynamic generalized linear model that accounted for time lags, overall trend, and seasonal variation. Our model showed an increase of leptospirosis cases associated with higher than expected rainfall, lower than expected temperature and higher than expected humidity. There was a lag of one-to-two weeks between weekly values for significant meteorological variables and leptospirosis incidence. Independent of the season, a weekly cumulative rainfall anomaly of 20 mm increased the risk of leptospirosis by 12% compared to a week following the expected seasonal pattern. Finally, over the 14-year study period, the annual incidence of leptospirosis decreased significantly by a factor of 2.7 (8.3 versus 3.0 per 100,000 people), independently of variations in climate.

**Conclusions:**

Strategies to control leptospirosis should focus on avoiding contact with contaminated sources of *Leptospira* as well as on increasing awareness in the population and health professionals within the short time window after low-level or extreme high-level rainfall events. Increased leptospirosis incidence was restricted to one-to-two weeks after those events suggesting that infectious *Leptospira* survival may be limited to short time intervals.

## Author summary

To determine the role of meteorological variables, seasonal variation and temporal trends in the incidence of leptospirosis, we investigated the time series of leptospirosis incidence amongst residents of Salvador, Brazil, from 1996 to 2010. Exploratory and confirmatory statistical methods detected associations between meteorological anomalies and disease incidence. Results showed the importance of extreme meteorological conditions, particularly rainfall, as short-term predictors of leptospirosis incidence. In addition, we found a long-term decreasing trend in disease incidence over the observation period.

## Introduction

Approximately one billion people worldwide reside in slums and this population continues to grow [1]. In these overcrowded environments with inadequate infrastructure, the burden of environmentally-transmitted infections such as helminths, bacterial diarrheal diseases and leptospirosis is growing and causes more than half a million deaths and 57 million Disability Adjusted Life-Years annually [2–6]. For these environmentally driven diseases, complex interactions of meteorological factors with inadequate sanitation and poor environment influence the transmission dynamics.

Urban leptospirosis epidemiology is an outstanding example of how the interaction between the environment and weather affects human health. The annual global burden of leptospirosis is estimated to be more than one million human cases and 58 thousand deaths [7]. A large fraction of this global burden falls on urban slum communities where people live in close proximity to animal and environmental reservoirs of infection [8]. In these settings, leptospirosis transmission is related to contamination of the peridomestic household environment [9–12] with urine from animal reservoirs, most notably rats [13,14].

Extreme weather events, including heavy rainfall and flooding, have been consistently associated with an increased incidence of urban leptospirosis [15–17]. In addition to these large sporadic events, seasonal leptospirosis patterns associated with cyclic rainfall have been described in several tropical developing countries [18–25]. Previous studies have suggested that, in addition to rainfall, other climatic factors, such as temperature and humidity may also independently contribute to leptospirosis transmission [18,20,26]. A recent study by Matsushita *et al* [27] describes correlations between relative moderate-to-low rainfall events and clinically suspected cases of leptospirosis. However, no study to date has disentangled the complex interactions of rain, temperature and humidity with the incidence of urban leptospirosis at short time lags. Long-term datasets obtained through prospective clinical laboratorial case ascertainment and analyzed at fine scale temporal resolution are needed to characterize the natural history of urban leptospirosis.

Our data illustrate the well-known fact that leptospirosis incidence is relatively high at times of year when rainfall, humidity and temperature are also expected to be relatively high. Here, we investigate the relationship between weekly laboratory confirmed leptospirosis incidence and meteorological anomalies, i.e. the residual values of temperature, rainfall and humidity after adjusting for their expected values. We analysed data from an active population-based surveillance system for leptospirosis in the Brazilian city of Salvador from 1996 to 2010. We used a dynamic generalized linear model [28] of incidence that took into account the autocorrelation structure of the weekly incidence series to analyze the time-lagged effects of meteorological anomalies on leptospirosis while taking account of long-term trend and seasonal variation.

## Methods

### Ethics statement

The study protocol received IRB approval from the Oswaldo Cruz Foundation (397/2010), the Brazilian National Commission for Ethics in Research (25000.071257/2010.57) and Yale University (1006006956). All adult participants provided written informed consent. Participants aged <18 years and who were able to read signed an informed assent, with their parents providing a signed consent. The study team used a standardized data entry form to interview the patients or a family member and to collect data from their medical charts after formal consent. Data included demographics, signs and symptoms at presentation and during hospital stay, clinical management, and disease outcome.

### Study site

Salvador, the fourth largest city in the country, is located in north-eastern Brazil and is the capital of the state of Bahia. In Brazil, notification of leptospirosis cases is mandatory. Over the study period, according to the State Secretary of Health’s protocol, patients suspected of leptospirosis are referred to the state infectious diseases hospital (Hospital Couto Maia) for diagnosis and clinical management. This hospital reported 75.4% of the 1,524 reported cases of leptospirosis among residents of Salvador between 2000 and 2010 [29].

### Surveillance

Since 1996 the Oswaldo Cruz Foundation, in collaboration with the Salvador and Bahia Secretaries of Health, has conducted uninterrupted enhanced hospital-based surveillance for leptospirosis in Salvador. Surveillance is primarily based at the state infectious diseases hospital where the study team prospectively evaluated admissions between March 21, 1996 to March 20, 2010 to identify cases who met the clinical definition for suspected severe leptospirosis, defined as a hospitalized patient with acute undifferentiated fever associated with either bleeding, acute renal failure, jaundice, or acute liver injury with transaminases <1,000 U/L [2,30].

### Laboratory diagnosis of leptospirosis

Study protocol included an early acute-phase blood sample collected at enrolment, a late acute-phase blood sample collected 2–7 days later, and a convalescent-phase blood sample collected more than 14 days after the first sample. Blood samples were processed, and sera frozen at -20°C and at -70°C. All sera underwent an IgM enzyme-linked immunosorbent assay (ELISA) (Biomanguinhos, Oswaldo Cruz Foundation, Brazil) and microagglutination test (MAT) using a serum panel comprising a local isolate–*Leptospira interrogans* serovar Copenhageni, strain Fiocruz L1-130 [2,31] and reference strains included in the WHO panel (*L*. Canicola H. Ultrecht IV, *L*. Autumnalis Akiyami A, *L*. Ballum Mus 127, *L*. Grippotyphosa Duyster, *L*. Cynopteri 3522C, *L*. Shermani LV 3954, *L*. Icterohaemorragiae RGA, *L*. Djasiman Djasiman, *L*. Icterohaemorragiae M20, *L*. Javanica Cox, *L*. Louisiania LSU1945, *L*. Panama CZ214K, *L*. Semaranga Patoc1, *L*. Sejroe Hardjoprajitno, *L*. Tarassovi Perepelitsin, *L*. Ballum Castellon 3, *L*. Bataviae Van Tienen, *L*. Sejroe 3705, *L*. Pyrogenes Salinem, *L*. Pomona Pomona, *L*. Celledoni Celledoni, *L*. Hebdomais Hebdomadis, *L*. Mini Sai, *L*. Shermani 1342K, *L*. Australis Jez Bratislava e *L*. Shermani LV 4135). *Leptospira* culture was also attempted from each acute-phase blood sample [2].

We defined a confirmed case of leptospirosis as one that met at least one of the following criteria: *Leptospira* isolation in blood culture, MAT seroconversion (defined as an increase in MAT titer from 0 to ≥1:200) or MAT four-fold titer rise between paired sera, MAT titer ≥1:800 in a single sample, or a positive result in the IgM-ELISA.Data-definitions.

As our outcome variable, we considered only laboratory-confirmed cases of leptospirosis. The exact date of admission of leptospirosis cases would give a spurious precision to the time of infection and onset of symptoms because of the imperfect link between infection, onset of symptoms, and hospital admission. We therefore aggregated the daily numbers cases to totals per study week, based on the registered date of onset of symptoms. The resulting time series spanned 733 weeks.

Various daily meteorological measurements were obtained from the meteorological station located in Ondina, Salvador and similarly aggregated into weekly values, includingaccumulated rainfall, average relative humidity and average maximum temperature. Three extreme values of rainfall were considered outliers and were truncated at 300mm, to guard against their disproportionately influencing results (the corresponding untruncated values were 402mm in April 1996, 378mm in March 2005 and 307mm in April 2009).

The population of Salvador was obtained from a population count in 1996 and from the censuses of 2000 and 2010 [32,33]. Population counts for the remaining study years were obtained by exponential interpolation.

### Statistical analysis

In an exploratory analysis to investigate patterns of overall trend and seasonality, separate working models were fitted to the time series of the transformed incidence, *Y*_*t*_ = log(incidence+1), and of the three meteorological variables: weekly accumulated rainfall, weekly average relative humidity, and weekly average of maximum temperature. For each variable, we calculated a residual series after fitting long-term trend and seasonal effects (S1 Text). Time-lagged associations between incidence and meteorological anomalies were then identified from cross-correlograms of these residual series.

To explore the relationship between transformed incidence and explanatory variables, we fitted non-parametric models for the regression of the transformed incidence residual on each of the meteorological residuals using generalized additive models [34].

For the confirmatory analysis, we then fitted a dynamic generalized linear model [28,35] in which weekly incidences are assumed to form an independent sequence of Poisson-distributed random variables conditional on a dynamic log-linear regression model. As regression terms we considered residual meteorological components lagged at 1 and 2 weeks together with dynamic, i.e. stochastically varying, trend and seasonal components to capture the trend, seasonal and residual correlation structure of the incidence series. The changing size of the population at risk over the study period was also included as an offset (S1 Text).

We used a Bayesian approach for the estimation of the model parameters, using integrated nested Laplace approximations [36] to perform the required calculations. We used the Deviance Information Criterion (DIC) to compare the fits of different models to our data [37]. For each one of these measures, there is no absolute benchmark for a well-fitting model, but in relative terms a smaller value represents a better fit.

We also used the fitted model to make one-step ahead predictions of incident case numbers, in each week holding the dynamic trend and seasonality terms fixed at their most recent inferred values. Analysis was done using *R* (R Development Core Team) [38] (S1 Text).

## Results

The surveillance team identified 2,074 clinically suspected cases of leptospirosis. We collected an acute sample for 1,666 (80.0%) and paired samples for 1032 (49.6%) of the suspected cases. Among clinically suspected cases with available samples, 1,473 (71.0%) were laboratory-confirmed. Laboratory confirmation was performed by MAT (1126; 76.4%), PCR (67; 4.5%), isolation (129; 8.8%) and IgM Elisa (162; 11.0%).

Laboratory-confirmed cases had a mean age of 35 years [Standard deviation (SD): 15], and 1266 (85.9%) were men. The mean period between onset of symptoms and hospital admission was 6 days (SD: 3). Clinical characteristics included conjunctival suffusion (519/1.131; 46%), jaundice (1293/1470;88%), renal failure defined by serum creatinine ≥ 1.5 mg/dL (597/689; 87%), and pulmonary haemorrhage with acute lung injury (40/798; 5%).

**Fig 1:**
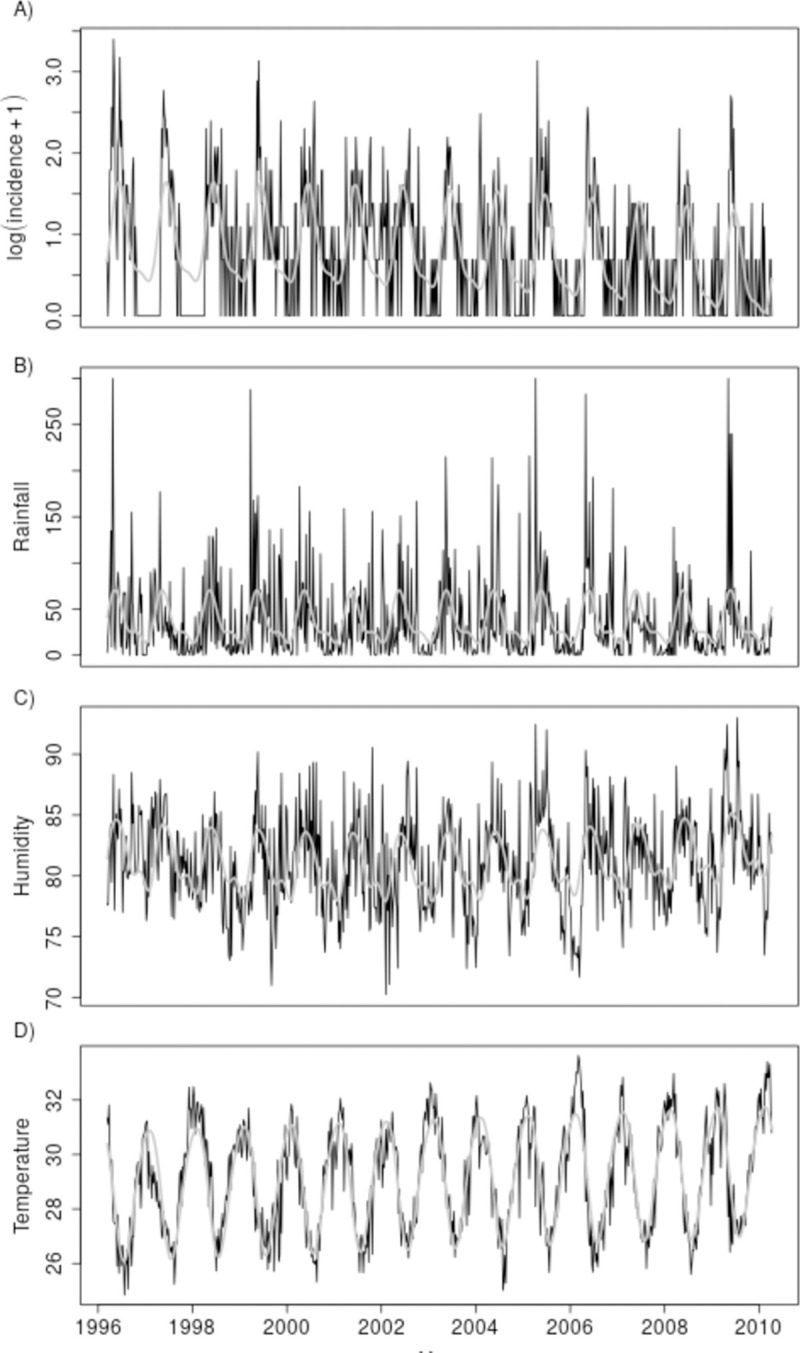
Observed time series (black) and fitted trends (grey) for transformed weekly laboratory confirmed leptospirosis incidence series (panel A) and meteorological time series: weekly accumulated rainfall in mm (panel B); weekly average of mean daily relative humidity in percent (panel C); and weekly average of mean daily maximum temperature in °C (panel D), Salvador, Brazil, 1996–2010.

The time series of weekly incidence shows a pronounced seasonal variation coupled with a decreasing trend (Fig 1A). A visual comparison of each of the meteorological time series with the transformed incidence series suggests that the periods of relatively high incidence of leptospirosis, which occur between April and July each year, coincide with periods of generally high rainfall, high relative humidity and low relative temperature (Fig 1B, 1C and 1D).

The cross-correlograms of the incidence residual series with each of the meteorological residual series confirm these associations. They show pronounced peaks (positive for rainfall and humidity, negative for temperature) at one-week and two-week time lags (S1 Fig). Examination of the fitted non-parametric regressions and their associated pointwise 95% confidence limits suggested only a weak departure from linearity between the transformed incidence residual and meteorological residual series (S2 Fig). Together, these results of the exploratory analysis justify our treatment of the one-week and two-week lagged meteorological variables as fixed effects in a log-linear model for incidence.

As a first step in the confirmatory analysis, we considered which of the three residual meteorological variables should be included in the dynamic model. Table 1 compares the DIC values for the eight sub-models that included each possible combination of the three pairs of one-week and two-week lagged residual meteorological variables. The first four lines show that rainfall was the most important meteorological variable associated with leptospirosis incidence. Adding either temperature or humidity gave substantial reductions in DIC. Including all three variables gave a further, albeit very slight, reduction in DIC and for this reason we selected the model including all three meteorological variables.

**Table 1:** Model fit from eight different combinations of the three pairs of meteorological explanatory variables to predict leptospirosis weekly incidence, Salvador, Brazil, 1996–2010.

Meteorological variables	DIC†
None	2274.8
(Rainfall_t-1_, Rainfall_t-2_)	2190.6
(Humidity_t-1_, Humidity_t-2_)	2234.4
(Temperature_t-1_, Temperature_t-2_)	2232.1
(Rainfall_t-1_, Rainfall_t-2_), (Humidity_t-1_, Humidity_t-2_)	2190.2
(Rainfall_t-1_, Rainfall_t-2_), (Temperature_t-1_, Temperature_t-2_)	2186.2
(Humidity_t-1_, Humidity_t-2_), (Temperature_t-1_, Temperature_t-2_)	2219.7
(Rainfall_t-1_, Rainfall_t-2_), (Humidity_t-1_, Humidity_t-2_), (Temperature_t-1_, Temperature_t-2_)	2185.9

Table 2 shows the estimated change in the relative risk associated with a unit change in each meteorological variable and associated 95% credible intervals. For example, a weekly cumulative rainfall residual of 20 mm increased the risk of leptospirosis by 12% compared to a week following the seasonal pattern. All the interval estimates excluded the neutral value 1, except for one-week-lagged residual temperature and humidity. However, we prefer to retain both one-week and two-week lagged terms because a model that included two-week lagged variable but excluded one-week lagged variable would be inconsistent with the known incubation period for the disease (5–14 days) [5]. Parameter values for the final model are described in S1 Table.

**Table 2:** Estimated changes in risk of leptospirosis, Salvador, Brazil, 1996–2010. Each row gives the estimate of the relative risk associated with a unit change in the corresponding meteorological variable and the associated 95% Bayesian credible interval.

Meteorological variable	Relative risk	Credible Interval (95%)
Rainfall_t-1_	1.006	(1.004;1.007)
Rainfall_t-2_	1.004	(1.003;1.006)
Humidity_t-1_	1.019	(0.997; 1.042)
Humidity_t-2_	1.027	(1.005; 1.049)
Temperature_t-1_	1.010	(0.917;1.113)
Temperature_t-2_	0.865	(0.787;0.950)

t-1 and t-2 correspond to time lag of one and two weeks, respectively.

**Fig 2:**
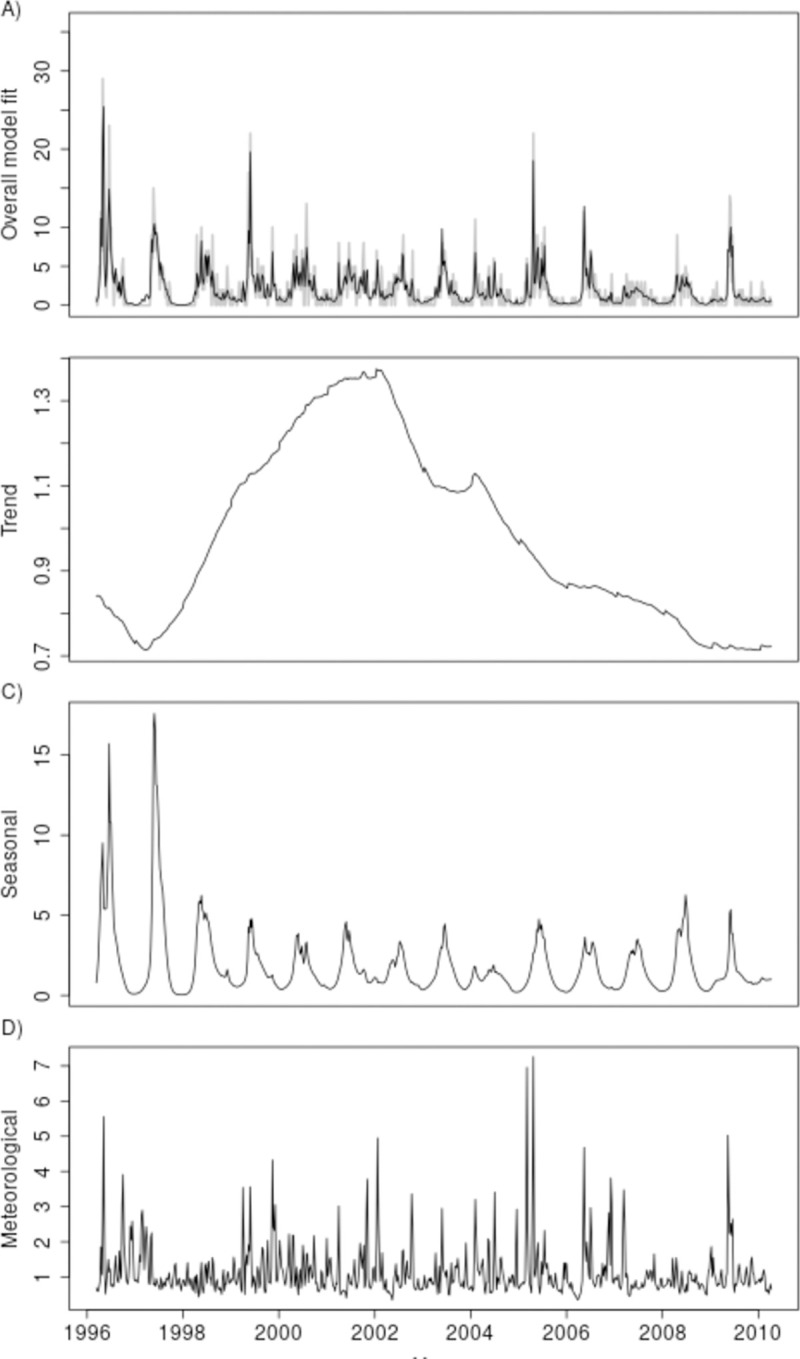
Panel A shows fitted weekly leptospirosis incidence (black solid line) and observed weekly laboratory confirmed leptospirosis incidence (grey solid line) and its respective decompositions into trend (panel B), seasonal (panel C) and meteorological (panel D) components, Salvador, Brazil, 1996–2010.

The expected weekly incidence according to the fitted model can be decomposed into trend, seasonal and meteorological multiplicative components (Fig 2). Together, these explained about 79.0% of the variation in the observed leptospirosis incidence. The fitted values closely followed the observed temporal distribution of leptospirosis cases (Fig 2A); 98.5% of the observed values were covered by the 95% credible intervals of the fitted model. The long-term trend throughout the observation period (Fig 2B) showed an increasing phase between 1998 and 2002 followed by a decreasing phase, with a peak around 1.3 cases per week between 2001 and 2003. These represent temporal changes in expected incidence that were not explained either by seasonal effects or by variation in the residual meteorological series. The seasonal component (Fig 2C) showed higher amplitudes in the early years and smaller year-to-year variations in amplitude and phase thereafter. The meteorological component (Fig 2D) demonstrated the importance of extreme meteorological conditions, showing relatively long periods close to one, interspersed with sharp peaks in incidence, representing up to a five-fold increase around extreme weather events. Meteorological variables explained 17.8% of the total variability in the data.

**Fig 3:**
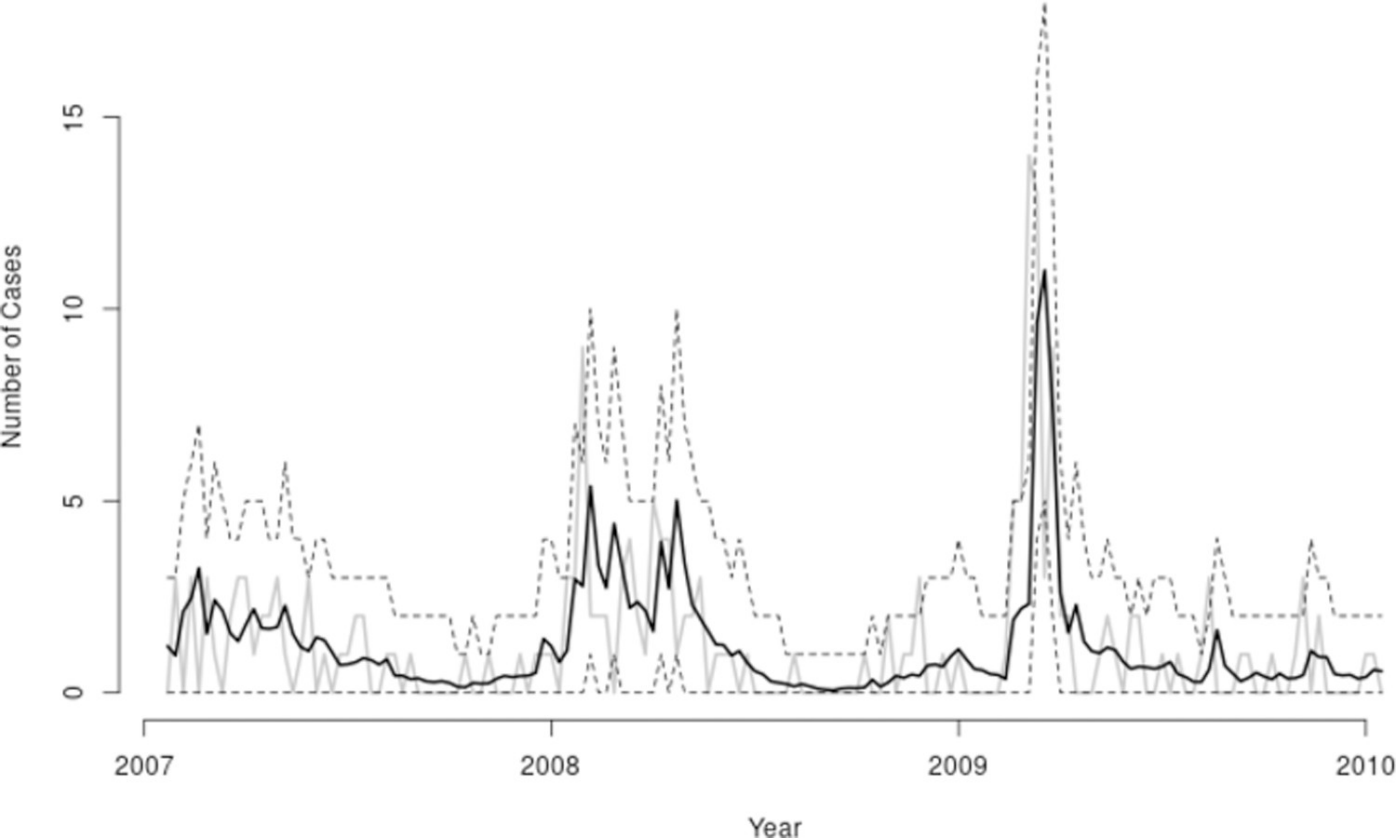
Observed weekly laboratory confirmed leptospirosis incidence (grey solid line), one-week-ahead forecasts (black solid line) and 95% credible intervals (black dashed lines) for the last three years (2007–2010) of the observations period in Salvador, Brazil.

The observed incidence series together with one-week-ahead point predictions and 95% credible intervals over the last three years (156 weeks) of the observation period are shown in Fig 3. The predictions were well calibrated, as the 95% credible intervals covered 94.9% of the actual values. Note that the credible intervals shown here were for observed rather than expected incidence, i.e. they included an allowance for Poisson variation in each observed count conditional on the underlying risk. Sensitivity analysis showed a relatively good performance for these predictions. For example, when the observed case numbers were zero, 79% and 93% of predicted values were less than 1 and 2, respectively, whereas when the number of observed cases was 4 or more, about 30% and 80% of the predicted values were greater than 3 and 2, respectively.

## Discussion

Leptospirosis is an emerging infectious disease problem in urban slums of developing tropical countries that is driven by two major factors: poverty and climate. This time-series analysis provides novel insights into the meteorological determinants of urban leptospirosis. Using weekly data points, we established and quantified a short-term association between leptospirosis incidence and meteorological variables while taking into account seasonal variation and long-term trend. We demonstrated that a combination of both relative low-level and extreme rainfall events, as well as temperature and humidity, influence leptospirosis incidence. Increased transmission risk was also restricted to one-to-two weeks after such events, suggesting that the survival of the infective pathogen in the environment may be limited to a short time interval. This increased understanding of the natural history of leptospirosis can aid in focusing control measures and support the development of infrastructure that will prevent small scale flooding.

The incidence of leptospirosis cases increased with both relative low levels of rainfall and extreme rainfall events. This was true even outside the rainy season. For example, a week with 20 mm higher rain than the expected level for that time of year increases the risk of the disease by 12%. While extreme rainfall events affect large areas [39–41], the impact of moderate rainfall on leptospirosis transmission may specifically reflect the precarious state of drainage systems in slum communities, which in turn may increase the risk for exposure to contaminated flood and sewage water. A study from urban Manila in the Philippines also identified that weekly rainfall (specifically at a two-week lag) was associated with increased hospital admission for leptospirosis, with the greatest risk after heavy to torrential rainfall [27]. Our data, together with the previous work in Manila, suggest that weekly intensity of rainfall may be a useful predictor of leptospirosis incidence, complementing previous approaches that use accumulated monthly values [20,42,43].

Our study identified positive short-term associations of rainfall with leptospirosis at lags of 1 and 2 weeks. Previous work in Manila identified that rainfall was associated with a higher number of clinically suspected leptospirosis cases at a lag time of 2 weeks [27]. Reasons for the shorter time lags found in our work may be explained by differences in methodology. Our study performed active case finding across the study period for ascertainment of clinical leptospirosis and confirmed cases with gold-standard laboratory testing. The study in Manila included suspected leptospirosis cases [27] with an expected rate of laboratory confirmation of 54% [44]. Correct estimates of this time lag between rainfall and disease incidence are important for accurate prediction of short-term leptospirosis risk and direction of public health interventions.

Our identification of one-to-two-week time lags is consistent with the expected incubation period plus the time until onset of illness, between 5 and 14 days [45]. The 1 to 2 weeks lag between rainfall, temperature, or humidity and leptospirosis identified here, as well as the 2 weeks lag between rainfall and flooding reported in Manila, contrast with results from previous studies that used monthly data and suggested temporal association between onset of symptoms of leptospirosis and meteorological data with lags between 2 and 10-months [18–20,46]. Although leptospires survive for up to one year and remain virulent in culture conditions that mimic the external environment *in vitro* [45,47,48], *L*. *interrogans* survival in spring water decreases to 3 weeks [49].

In our analysis, previous weekly temperatures and humidity were negatively and positively associated, respectively, with leptospirosis incidence. Seasonal variations in rainfall and humidity were inversely related to seasonal temperature variation. A similar relationship was found in Reunion Island [20]. Although the meteorological variables in this study are moderately correlated (r = 0.48, for humidity and rainfall) our results suggest that these variables have independent effects on disease risk. Both lower temperatures and higher levels of humidity could increase bacterial environmental survival by slowing leptospire desiccation, [45] in turn increasing the risk for human leptospirosis [50]. Salvador is a coastal city and its relative humidity is dependent on a diversity of factors including water evaporation from the Atlantic Ocean. This additional source of humidity could be important for leptospirosis survival in the environment.

Maximum temperature in Salvador varied between 26 and 32°C (Fig 1); changes within this range could affect leptospire survival given that, under laboratory conditions, best growth is obtained between 28–30°C [20,45]. Our findings differ from results obtained in Reunion Island and Thailand, where temperature was positively associated with leptospirosis incidence [18,20]. However, both of these data sets used mean temperature as opposed to maximum and, in Thailand, months with higher temperatures also have higher rainfall. In Salvador, where higher temperatures are registered during the drier months, increased maximum temperature likely increases desiccation, so decreasing leptospire viability in the environment.

Limited evaluation of statistical approaches is available for the study of associations between meteorological factors and leptospirosis or other water-associated diseases [51]. Previously, leptospirosis incidence has been modeled with ARIMA models [18,20]. Whereas ARIMA require a preliminary transformation of the data to achieve stationarity, the dynamic model used in this study can be applied to non-stationary time series without requiring a preliminary transformation of the data. Recently, data from Manila in the Philippines considered distributed lag non-linear models (DLNM) to evaluate the nonlinear temporal dependence between the incidence of leptospirosis and rainfall levels allowing for time delayed effects [27]. The dynamic model used here also accounts for possible nonlinear and lags of time effects between meteorological variables and leptospirosis incidence rates. Furthermore, the dynamic model considers the intrinsic temporal correlations amongst weekly incidences, so that we can be more confident about the validity of our inferences. Additionally, the proposed model makes it possible to evaluate separately the effects for trend, seasonality, and meteorological variables.

We also identified a long-term temporal increase and decline in case incidence that was independent of variations in climate. The modeling approach enabled an evaluation of meteorological effects independently of the cumulative long-term influence of interventions designed to reduce disease risk over the study period. There was a significant decline in incidence of severe leptospirosis during the 14-year period with annual incidence 2.7-fold lower in 2009 than in 1996 (3.0 vs. 8.3 per 100,000 population). This decline did not appear to be due to decreased case ascertainment or change in referral patterns. However, a major intervention was made in the sanitation infrastructure of Salvador during the surveillance period. A $440 million Inter-American Development Bank-supported program (Bahia Azul) constructed 2,000km of closed sewage systems between 1996 and 2004 [52], increasing the city’s population served by safe sewage connections from 26% to 74% [53]. A prospective study demonstrated that the program led to a 22% reduction in diarrheal disease prevalence [52]. Additionally, during 1995–2006 the increase in gross domestic product and social conditional cash transfer programs for the poorest populations in Brazil contributed to reducing poverty and marginally decreasing social and economic inequalities [54]. These actions to reduce poverty may have influenced the observed decrease of leptospirosis incidence. Additionally, changes in seasonality of non-meteorological drivers of transmission, as rat reservoir abundance, could influence the general decline on leptospirosis incidence, however the lack of information at city level prevented further analysis of those drivers.

Correlation of seasonal variations in leptospirosis disease with temperature and rainfall does not establish a causal link. Also, we did not include information relating to the spatial distribution of leptospirosis incidence. 11% of cases were confirmed by Elisa, which is not gold-standard for leptospirosis confirmation. However, those patients met the criteria for clinical leptospirosis and no other pathogens were identified. Our model is related to surveillance of cases and meteorological events within urban Salvador, Bahia and has not been tested for generalizability to other locations or patterns of disease transmission. However, 28% of the Brazilian population live in urban slums [55] with environmental and socioeconomic conditions similar to the slums of Salvador. These figures are comparable to other tropical developing countries [56]. Consequently, we believe that our results could be used to inform predictive modelling, with appropriate local recalibration, in many other urban communities.

This paper highlights important implications for the design of interventions against leptospirosis infection in urban slum communities. We have found that increased leptospirosis risk is associated with small amounts of rainfall deviations from the seasonal pattern in the last two weeks. This supports the taking of precautionary measures during any rainy period deviating from the seasonal pattern. Sanitation should be improved in vulnerable communities to prevent even small-scale flooding events. Moreover, we have identified that the period of raised infection risk appears to be during or in the immediate 2 weeks following a rain event. Our models provide useful evidence on the additional contribution of meteorological data to improving early warning systems, and increasing population and medical awareness within this short time window.

## Supporting information

S1 Data Excel spreadsheet containing, in separate spreadsheets, the data used in tables and figures. (CSV)

S1 Text Technical appendix for the statistical analysis. (DOCX)

S1 Table Values of final model parameters (Table 2) and their credible intervals. (DOCX)

S1 Fig Cross-correlograms of residuals of transformed leptospirosis incidence with residuals of meteorological variables: weekly accumulated rainfall, weekly average of mean daily relative humidity and weekly average of mean daily maximum temperature, Salvador, Brazil, 1996–2010. (TIFF)

S2 Fig Generalized additive models for the regression of transformed incidence residuals on weekly accumulated rainfall (left-hand panels), weekly average of mean daily relative humidity (center panels) and weekly average of mean daily maximum temperature (right-hand panels), at one-week (upper row) and two-week (lower row) time-lags, Salvador, Brazil, 1996–2010. The small bars on the horizontal axes show the individual values of each meteorological variable.
(TIFF)
