# Supplementary material for: Rainfall and other meteorological factors as drivers of urban transmission of leptospirosis
Source: PLoS Negl Trop Dis. 2022 Apr 11;16(4):e0007507. doi: 10.1371/journal.pntd.0007507 (PMC9022820; doi:10.1371/journal.pntd.0007507)
Supplement: S1 Text — (DOCX) [file pntd.0007507.s002.docx]

**S1. Text**

**Technical Appendix**

Exploratory analysis

Let $y_{t}$ denote the number of leptospirosis cases in wee$k t=1,\ldots,n$. In a preliminary descriptive analysis, we subtracted long-term trend and seasonal effects from the time series of $Y_{t}=\log\left( 1+y_{t} \right)$and of the meteorological variables: cumulative rainfall ($R_{t}$); humidity ($H_{t}$); and maximum temperature ($T_{t}$). For each of these four variables, the working model for the trend, $\mu_{t}$, was

$$\mu_{t}=\beta_{0}+\beta_{1}t+\beta_{2}t^{2}+\alpha_{1}\cos\left( 2\pi t/52.14 \right)+\alpha_{2}\sin\left( 2\pi t/52.14 \right)+\alpha_{3}\cos\left( 4\pi t/52.14 \right)+\alpha_{4}\sin\left( 4\pi t/52.14 \right),$$

with parameters estimated separately for each variable.

Model Formulation

Our proposed model is a dynamic generalized linear model [1, 2][3West and Harrison, 1997), in which weekly incidences,$y_{t}$, are assumed to form an independent sequence of Poisson-distributed random variables conditional on a log-linear regression model with one-week and two-week time-lagged versions of the three residual meteorological time series as explanatory variables, and dynamic regression coefficients for a time-trend and seasonal variation in adjusted incidence, in each case modelled as random walks. Hence,

$$y_{t}|\theta_{t}\sim Poisson\left( \theta_{t} \right):t=1,\ldots,n (1)$$

$$\log\left( \theta_{t} \right)=\text{log}\left( {Pop}_{t} \right)+M_{t}+S_{t}$$

$$M_{t}=B_{t}+\beta_{1}R_{t-1}+\beta_{2}R_{t-2}+\beta_{3}H_{t-1}+\beta_{4}H_{t-2}+\beta_{5}T_{t-1}+\beta_{6}T_{t-2}$$

$$S_{t}=A_{1,t}\sin\left( \frac{2\pi t}{52.14} \right)+A_{2,t}\cos\left( \frac{2\pi t}{52.14} \right)$$

$$B_{t}=B_{t-1}+U_{t}$$

$$A_{j,t}=A_{j,t-1}+W_{j,t}, j=1,\ldots,2$$

$$U_{t}\sim iid N\left( 0,\vartheta^{2} \right)$$

$$W_{j,t}\sim iid N\left( 0,\tau_{j}^{2} \right)$$

Note that the estimated expected weekly incidence is the product of three components: $\text{trend=exp}\left( \hat{\beta}_{t} \right)$, $\text{seasonal=exp}\left( \hat{A}_{1,t}\sin\left( 2\pi t/52.14 \right)+\hat{A}_{2,t}\cos\left( 2\pi t/52.14 \right) \right)$and m$\text{eteorological=exp}\left( \hat{\beta}_{1}R_{t-1}+\hat{\beta}_{2}R_{t-2}+{\hat{\beta}_{3}H_{t-1}+\hat{\beta}_{4}H_{t-2}+\hat{\beta}}_{5}T_{t-1}+\hat{\beta}_{6}T_{t-2} \right).$

Model selection

To compare the fits of different models to our data, we use the Deviance Information Criterion [3]. A second measure of fit is a cross-validated logarithmic score [4] a type of “leave-one-out'' predictive measure; in this case, there is no absolute benchmark for a well-fitting model, but in relative terms a smaller score represents a better fit.

**References**

1. West M, Harrison J. Bayesian Forecasting and Dynamic Models. 2nd ed: Springer; 1997.

2. West M, Harrison P, Migon H. Dynamic generalized linear models and Bayesian forecastin. Journal of the American Statistical. 1985;80(389):73-83. doi: 10.1080/01621459.1985.10477131

3. Spiegelhalter DJ, Best NG, Carlin BP, van der Linde A. Bayesian measures of model complexity and fit. Journal of the Royal Statistical Society: Series B (Statistical Methodology). 2002;64:583-639. doi: 10.1111/1467-9868.00353. PubMed PMID: 6175412381464386404.

4. Gneiting T, Raftery AE. Strictly Proper Scoring Rules, Prediction, and Estimation. Journal of the American Statistical Association. 2007;102(477):359-78. doi: 10.1198/016214506000001437.

R analysis

#Data preparation

library(MASS)

library(epitools)

library(gdata)

library(gtsummary)

library(dplyr)

##############################################################

casosnew <- read.csv2("casos.csv",sep=";")

casosnew1 <- read.csv("geocases.csv")

names(casosnew1)[c(1,2,18)]<-c("vigi","date","dataini")

casosnew1$DATA <- as.Date(casosnew1$date, "%d/%m/%Y")

casosnew1$DATAINI <- as.Date(casosnew1$dataini, "%d/%m/%Y")

casosnew1$juliano <- julian(casosnew1$DATAINI,origin=as.Date("1995-12-31"))

casosweek <- as.week(casosnew1$DATAINI)

casosnew1$week <- casosweek$week

aux<-order(casosnew1$DATAINI)

casosnew1<-casosnew1[aux,]

aux<-is.na(casosnew1$DATAINI)

casosnew1<-casosnew1[!aux,]

inmetnew <- read.csv2("dados_chuva.csv",sep=";",header=T)

inmetnew1 <- read.csv("clima.csv")

with(inmetnew,plot(PLUV,type="l", xaxt="n", ylab="Chuva", xlab=""))

with(inmetnew,plot(UMID,type="l", xaxt="n", ylab="Umidade", xlab=""))

with(inmetnew,plot(TMAX,type="l", ylab="Temperatura", xlab=""))

inmetnew$DATA <- as.Date(inmetnew$DATA, "%d/%m/%Y")

inmetnew$ANO<-as.numeric(getYear(inmetnew$DATA))

inmetnew$MES<-as.numeric(getMonth(inmetnew$DATA))

inmetnew$juliano <- julian(inmetnew$DATA,origin=as.Date("1995-12-31")) #n. dias desde a origem

climaweek <- as.week(inmetnew$DATA) #numero da semana no ano (para cada ano, varia de 1 a 52)

inmetnew$week <- climaweek$week

inmetnew$SE <- c(rep(1,6),rep(2:745, each=7), 746) #numeracao das semanas 1,..1(7vezes),2,...,2(sete vezes),...

leptonew <- merge(inmetnew,casosnew1,by="juliano", all.x=T, all.y=T)

leptonew$CASO <- NA

#select only confirmed cases

leptonew$CASO<-NA

leptonew$CASO[leptonew$confirm==0]<-0

leptonew$CASO[leptonew$confirm==1|leptonew$confirm==2]<-1

minNA <- function (x) { min(x,na.rm=T)}

meanNA <- function (x) { mean(x,na.rm=T)}

sumNA <- function (x) {sum(x,na.rm=T)}

leptonew.se= NULL

leptonew.se$SE = 1:746

leptonew.se$ANO = aggregate(leptonew[,"ANO"], by=list(leptonew$SE),FUN=c("minNA"))$x

leptonew.se$MES = aggregate(leptonew[,"MES"], by=list(leptonew$SE),FUN=c("minNA"))$x

leptonew.se$PLUV = aggregate(leptonew[,"PLUV"], by=list(leptonew$SE),FUN=c("meanNA"))$x

leptonew.se$TPLUV = aggregate(leptonew[,"PLUV"], by=list(leptonew$SE),FUN=c("sumNA"))$x

leptonew.se$UMID = aggregate(leptonew[,"UMID"], by=list(leptonew$SE),FUN=c("meanNA"))$x

leptonew.se$TMAX = aggregate(leptonew[,"TMAX"], by=list(leptonew$SE),FUN=c("meanNA"))$x

leptonew.se$TMED = aggregate(leptonew[,"TMED"], by=list(leptonew$SE),FUN=c("meanNA"))$x

leptonew.se$TMIN = aggregate(leptonew[,"TMIN"], by=list(leptonew$SE),FUN=c("meanNA"))$x

leptonew.se$juliano = aggregate(leptonew[,"juliano"],by=list(leptonew$SE), FUN=c("min"))$x

leptonew.se$CASOS = aggregate(leptonew[,"CASO" ], by=list(leptonew$SE),FUN=c("sumNA"))$x

leptonew.se=data.frame(leptonew.se)

lepto.se <- leptonew.se[leptonew.se$SE<=743,]

lepto.se$CASOS[lepto.se$SE<=10] <- NA

lepto.se$CASOSC[lepto.se$SE<=10] <- NA

lepto.se$PLUV <- round(lepto.se$PLUV*7,0)

####example rainfall correlogram

ccf(rpluv,rcasos,main="Rainfall Residuals",cex.main=0.3)

#example gam descriptive analysis (rainfall)

library(mgcv)

lixo<-(data.frame(lagged(rpluv,1)[-1],rcasos[-1]))

colnames(lixo)<-c("chuva","casos")

ct1<-gam(casos~s(chuva),data=lixo)

ct1

plot(ct1,shade=TRUE,seWithMean=TRUE,scale=0,ylab="Incidence residuals",

xlab="Rainfall Residuals (lag=1)")

## removing seasonality (example rainfall)

pluv_t_1<-lepto.se$PLUV1[10:(nrow(lepto.se)-1)]

t=1:length(pluv_t_1)

s = c = matrix(nr=length(t), nc=6)

for (i in 1:6)

{ c[,i] = cos(2*pi*i*t/52.14)

s[,i] = sin(2*pi*i*t/52.14) }

model = lm(pluv_t_1~s+c)

summary(model)

model = lm(pluv_t_1~s[,1]+s[,2]+c[,1]+c[,2])

summary(model)

R_t_1<-resid(model)

length(R_t_1)

pluv_t_2<-lepto.se$PLUV1[9:(nrow(lepto.se)-2)]

t=1:length(pluv_t_2)

s = c = matrix(nr=length(t), nc=6)

for (i in 1:6)

{ c[,i] = cos(2*pi*i*t/52.14)

s[,i] = sin(2*pi*i*t/52.14) }

model = lm(pluv_t_2~s+c+t)

summary(model)

model = lm(pluv_t_2~s[,1]+s[,2]+c[,1]+c[,2])

summary(model)

R_t_2<-resid(model)

#Data preparation

n<-nrow(lepto.se)-10

SIN<-sin(2*pi*(1:n)/52.14)

COS<-cos(2*pi*(1:n)/52.14)

SIN2<-sin(2*2*pi*(1:n)/52.14)

COS2<-cos(2*2*pi*(1:n)/52.14)

y<-lepto.se$CASOS[11:(nrow(lepto.se))]

yc<-lepto.se$CASOSC[11:(nrow(lepto.se))]

ano<-lepto.se$ANO[11:(nrow(lepto.se))]

id <- id1 <- id2 <-id3<-id4<-id5<-id6<-id7<-id8<-id9<- 1:n

lepto.data<-data.frame(id,id1,id2,id3,id4,id5,id6,id7,id8,SIN,COS,SIN2,COS2,R_t_1,R_t_2,H_t_1,H_t_2,T_t_1,T_t_2,y,yc,ano)

head(lepto.data)

tail(lepto.data)

id <- id1 <- id2 <-id3<-id4<-id5<-id6<-id7<-id8<-id9<- 1:n

##Modelling

require(INLA)

formula <- y ~ f(id, model="rw1",initial=-0.1,constr=F) +

f(id1, SIN, model="rw1",initial=0.79,constr=F)+

f(id2, COS, model="rw1",initial=0.16,constr=F)+

f(id3, SIN2, model="rw1",initial=0,constr=F)+

f(id4, COS2, model="rw1",initial=0,constr=F)+

R_t_1+R_t_2+H_t_1+H_t_2+T_t_1+T_t_2-1

require(INLA)

r1 = inla(formula, family="poisson", data = lepto.data,control.predictor=list(compute=TRUE),control.compute=list(dic=1,cpo=1))

summary(r1)

dic1<-r1$dic[[1]]

lsr1 <- -mean(log(r1$cpo)[is.finite(log(r1$cpo))])

lsr1

# Other models can be found by chance covariates, trends, and seasonal terms in the formula.

#For predictions

formula <- y ~ f(id, model="rw1",initial=-0.1,constr=F) +

f(id1, SIN, model="rw1",initial=0.79,constr=F)+

f(id2, COS, model="rw1",initial=0.16,constr=F)+

R_t_1+R_t_2+H_t_1+H_t_2+T_t_1+T_t_2-1

prev<-NULL

for(i in 578:733){

lepto.prev<-lepto.data[1:i,]

naux<-nrow(lepto.prev)

lepto.prev$y[naux]<-NA

r_pred = inla(formula, family="poisson", E=pop,data = lepto.prev, control.predictor=list(compute=TRUE))

aux<-exp(r4_pred$summary.fitted.values[naux,1])*lepto.prev$pop[naux]

}

prev<-c(prev,aux)

print(i)

}

ic1<-NULL

ic2<-NULL

plot.ts(prev)
