# Supplementary material for: Rainfall and other meteorological factors as drivers of urban transmission of leptospirosis
Source: PLoS Negl Trop Dis. 2022 Apr 11;16(4):e0007507. doi: 10.1371/journal.pntd.0007507 (PMC9022820; doi:10.1371/journal.pntd.0007507)
Supplement: S1 Table — (DOCX) [file pntd.0007507.s003.docx]

**Supplemental Table 1:** Values of final model parameters (Table 2) and their credible intervals.

| **Fixed Effects** | **Parameter** | **Credible interval (95%)** |
| --- | --- | --- |
| $\beta_{1}$ | 0.0058 | (0.0043; 0.0073) |
| $\beta_{2}$ | 0.0044 | (0.0030; 0.0058) |
| $\beta_{3}$ | 0.0190 | (-0.0031; 0.0410) |
| $\beta_{4}$ | 0.0264 | (0.0046; 0.0481) |
| $\beta_{5}$ | 0.0104 | (-0.0868; 0.1077) |
| $\beta_{6}$ | -0.1452 | (-0.2392; -0.0512) |
| **Random effects** |  |  |
| ${1/\vartheta}^{2}$ | 2117.056 | (243.450; 8055.89) |
| ${1/\tau}_{1}^{2}$ | 9.397 | (5.399; 15.64) |
| ${1/\tau}_{2}^{2}$ | 3788.494 | (566.044; 12963.66) |
