# Supplementary figures and images for: Rainfall and other meteorological factors as drivers of urban transmission of leptospirosis
Source: PLoS Negl Trop Dis. 2022 Apr 11;16(4):e0007507. doi: 10.1371/journal.pntd.0007507 (PMC9022820; doi:10.1371/journal.pntd.0007507)

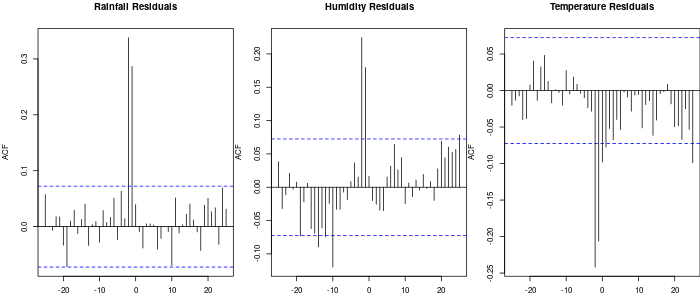

Supplement: S1 Fig — (TIFF) [file pntd.0007507.s004.tiff]

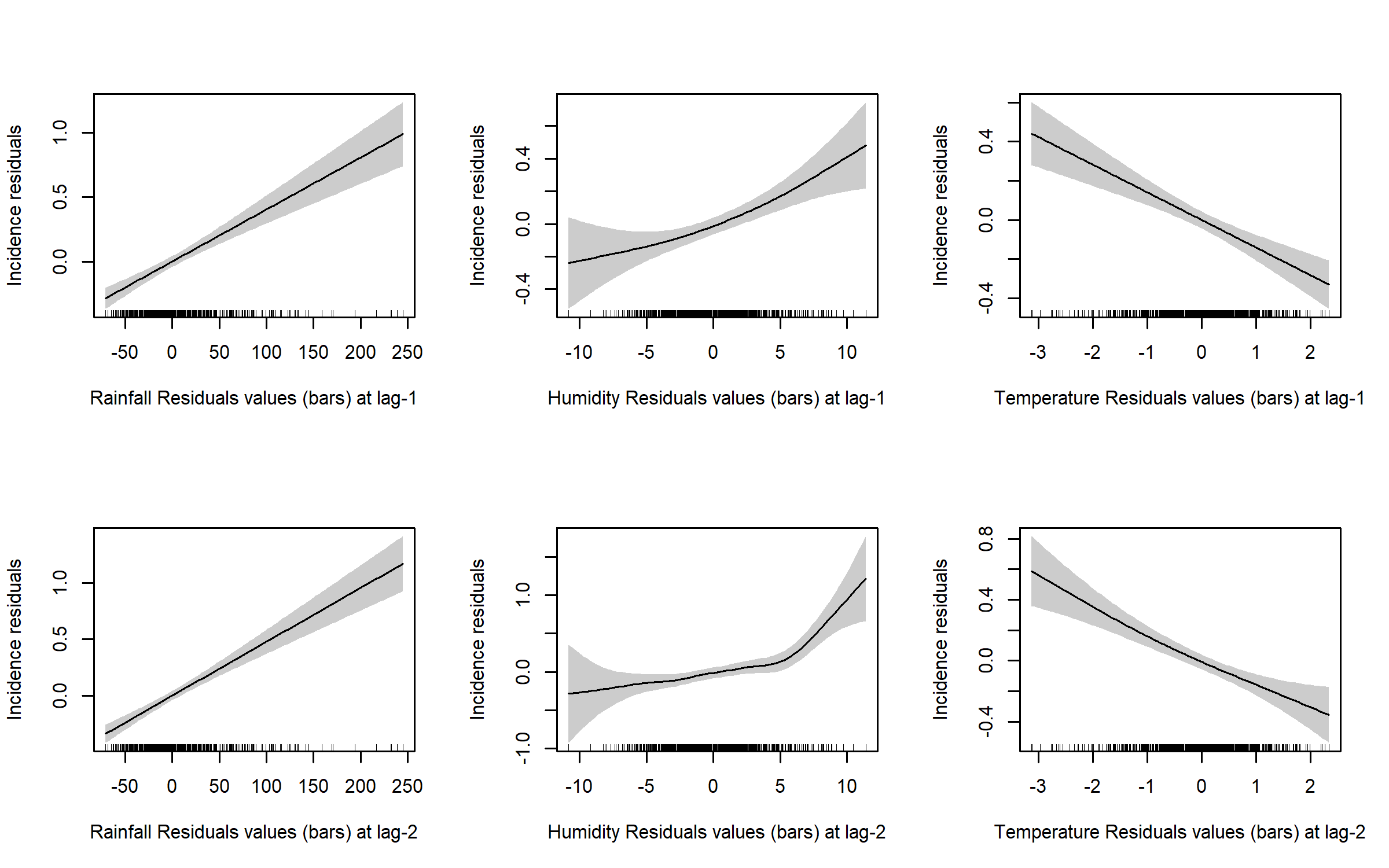

Supplement: S2 Fig — The small bars on the horizontal axes show the individual values of each meteorological variable. (TIFF) [file pntd.0007507.s005.tiff]
